# Guangdong's experience in defeating the COVID-19

**DOI:** 10.1097/MD.0000000000025881

**Published:** 2021-05-07

**Authors:** Haiqian Chen, Leiyu Shi, Yuyao Zhang, Xiaohan Wang, Gang Sun

**Affiliations:** a Department of Health Management, School of Health Management, Southern Medical University, Guangzhou, Guangdong, PR China; b Department of Health Policy and Management, Bloomberg School of Public Health, Johns Hopkins University, Baltimore, MD.

**Keywords:** COVID-19, epidemic control policy, Guangdong Province

## Abstract

To provide valuable experience for other countries currently fighting the COVID-19 pandemic by systematically analyzing the effects of control policies on coping with COVID-19 in Guangdong Province of China.

Integrating qualitative and quantitative methods to systematically analyze the effects of control policies on fighting COVID-19 with official data in Guangdong Province.

Between January 19, 2020 and February 26, 2020, Guangdong Province issued 6 critical control policies to cope with COVID-19 and achieved effects in the following 3-phase.

1. Phase 1: from January 19 to February 7, 2020, Guangdong Province issued the following 4 policies: activating the first-level response to public health emergencies; holding press conferences during the epidemic; carrying out grid investigation; and taking the lead in covering the treatment expenses of suspected patients in basic medical insurance. In this phase, the number of confirmed cases increased rapidly and the number of recovered cases increased gradually.
2. Phase 2: from February 8 to 14, Guangdong Province issued the following 2 polices: applying Toujie Quwen granules to 30 designated hospitals and launching a registration and report system for the sale of fever and cough drugs. In this phase, the growth trend of confirmed cases had slowed down on February 10 and then increased slowly on February 14. The number of recovered cases increased rapidly on February 10 and then increased rapidly on February 14.
3. Phase 3: from February 15 to 26, the increase number of confirmed cases was a small in magnitude on February 20 and then increased slowly on February 26. The number of recovered cases increased rapidly on February 20 and then increased rapidly on February 26.

Guangdong Province implemented 6 control policies in 3-phase and finally successfully defeated the COVID-19. In the first phase, the first 4 control policies showed initial effects on COVID-19 epidemic control. In the second phase, the last 2 control policies greatly contributed to containing the COVID-19 epidemic. In the third phase, the 6 control policies completely overcame the COVID-19 in Guangdong Province, China.

## 1 Introduction

Coronavirus Disease 2019 (COVID-19) is spreading worldwide, which has a great impact on the global politics, economics, and culture. In late December 2019, a number of COVID-19 cases were confirmed in Wuhan city and rapidly spread throughout China. Guangdong Province, with 1018 confirmed cases by February 6, 2020, had the second largest number of COVID-19 cases in China.^[1]^ To control the spread of the COVID-19 epidemic, Guangdong Province issued a series of critical control policies and achieved remarkable effects. This study systematically analyzed the effects of these policies on coping with COVID-19 in Guangdong Province, and provided valuable experience for global currently fighting the COVID-19 pandemic.

## 2 General situation of Guangdong province

Guangdong Province is a coastal province with the most populous in southern China and the economic aggregate of Guangdong Province ranks the first and account for 12.5% of the national total since 1989. Guangzhou is the capital of Guangdong Province with the total investment of enterprises ranks the top 3 in China. Shenzhen is the first special economic zone and its economic aggregate has long ranked the fourth in mainland China. Both Shenzhen and Guangzhou, which are the highest population density in China.^[2–4]^

## 3 The situation of COVID-19 in Guangdong province

### 3.1 Definitions

COVID-19 was diagnosed on the basis of the guidelines on novel coronavirus diagnosis and treatment. Those with one of the following pathogenic evidence is the confirmed case:

1. Positive for the 2019-nCoV by the real-time PCR test for nucleic acid in respiratory or blood samples.
2. Viral gene sequencing shows highly homogeneity to the known 2019-nCoV in respiratory or blood samples.

And the standards of the recovered cases were that the body temperature returned to normal for more than 3 days; respiratory symptoms improved significantly; inflammation of the lungs showed obvious signs of absorption; and respiratory nucleic acid was negative for 2 consecutive times (1-day sampling time interval at least); and the patient can be released from isolation.^[5,6]^

### 3.2 Overall situations

Tables 1 and 2 show as of February 26, 2020, the number of cumulative confirmed cases was 1347 and the number of cumulative recovered cases was 873 in Guangdong Province. Among them, Guangzhou and Shenzhen had the largest cumulative confirmed cases and cumulative recovered cases of COVID-19, accounting for more than 50% of the total Guangdong Province. In Guangzhou, the number of cumulative confirmed cases was 346 and cumulative recovered cases was 230. In Shenzhen, the number of cumulative confirmed cases was 417 and cumulative recovered cases was 271.

**Table 1:** The cumulative confirmed cases of COVID-19 in various cities of Guangdong Province (case).

Date	Guangzhou	Shen zhen	Zhu hai	Fo shan	Dong guan	Zhongshan	Hui zhou	Shan wei	Jiangmen	Zhan jiang	Zhao qing	Mei zhou	Yang jiang	Mao ming	Qing yuan	Shao guan	Jie yang	Shan wei	Chao zhou	He yuan	Yunfu	Total
2020/1/19	0	1	0	0	0	0	0	0	0	0	0	0	0	0	0	0	0	0	0	0	0	1
2020/1/20	0	10	3	0	0	0	0	0	0	1	0	0	0	0	0	0	0	0	0	0	0	14
2020/1/21	2	14	4	1	0	0	1	0	0	2	0	0	0	0	0	2	0	0	0	0	0	26
2020/1/22	5	15	4	1	0	1	1	0	0	2	1	0	0	0	0	2	0	0	0	0	0	32
2020/1/23	7	15	8	6	0	2	5	0	0	2	1	0	3	0	1	3	0	0	0	0	0	53
2020/1/24	14	20	10	9	1	2	7	1	0	2	2	0	5	0	2	3	0	0	0	0	0	78
2020/1/25	14	27	10	10	2	2	8	2	0	4	4	0	6	0	3	3	3	0	0	0	0	98
2020/1/26	39	36	12	14	2	4	8	2	0	5	4	1	8	0	3	4	3	1	0	0	0	146
2020/1/27	51	49	12	18	2	6	11	5	0	5	4	3	8	0	5	4	4	1	0	0	0	188
2020/1/28	63	63	14	18	7	12	12	6	0	7	5	4	10	2	6	4	6	1	0	1	0	241
2020/1/29	79	86	18	25	7	16	17	12	1	9	5	5	10	3	6	4	6	1	0	1	0	311
2020/1/30	106	110	26	32	16	18	17	12	1	11	6	5	10	3	6	4	6	3	0	1	0	393
2020/1/31	137	170	38	38	21	18	20	14	3	13	6	5	10	3	6	4	6	4	3	1	0	520
2020/2/1	175	196	41	39	27	21	23	14	3	14	7	6	10	3	6	5	6	4	3	1	0	604
2020/2/2	189	226	51	43	31	25	28	17	4	14	7	7	10	4	6	5	6	5	4	1	0	683
2020/2/3	216	269	64	46	37	31	29	18	6	15	10	9	12	5	8	6	6	5	4	1	0	797
2020/2/4	237	289	69	49	44	35	31	20	7	18	11	10	12	6	10	6	6	5	4	1	0	870
2020/2/5	255	314	73	50	47	44	35	20	9	19	13	12	13	6	10	6	7	5	5	1	0	944
2020/2/6	284	334	73	57	53	46	39	22	11	19	14	12	13	6	10	6	7	5	5	2	0	1018
2020/2/7	298	351	82	60	56	46	41	25	14	20	14	13	13	6	10	6	7	5	5	3	0	1075
2020/2/8	304	364	83	65	58	51	47	25	17	21	14	13	13	9	10	6	7	5	5	3	0	1120
2020/2/9	313	368	85	68	60	56	50	25	19	21	14	13	13	10	10	6	7	5	5	3	0	1151
2020/2/10	317	375	86	70	62	58	53	25	20	21	15	13	13	11	10	7	8	5	5	3	0	1177
2020/2/11	323	386	87	78	67	62	54	25	21	21	16	13	13	13	12	7	8	5	5	3	0	1219
2020/2/12	327	391	89	81	70	64	55	25	22	21	16	13	13	13	12	8	8	5	5	3	0	1241
2020/2/13	328	400	91	82	73	64	56	25	22	22	17	14	13	13	12	8	8	5	5	3	0	1261
2020/2/14	335	406	95	84	81	65	58	25	22	22	17	15	13	13	12	10	8	5	5	3	0	1294
2020/2/15	338	414	96	84	89	65	58	25	23	22	17	16	13	13	12	10	8	5	5	3	0	1316
2020/2/16	339	415	97	84	89	66	58	25	23	22	17	16	13	14	12	10	8	5	5	4	0	1322
2020/2/17	339	416	98	84	91	66	59	25	23	22	18	16	13	14	12	10	8	5	5	4	0	1328
2020/2/18	339	416	98	84	91	66	62	25	23	22	18	16	13	14	12	10	8	5	5	4	0	1331
2020/2/19	339	416	98	84	92	66	62	25	23	22	18	16	13	14	12	10	8	5	5	4	0	1332
2020/2/20	339	416	98	84	93	66	62	25	23	22	18	16	13	14	12	10	8	5	5	4	0	1333
2020/2/21	343	417	98	84	93	66	62	25	23	22	19	16	13	14	12	10	8	5	5	4	0	1339
2020/2/22	345	417	98	84	94	66	62	25	23	22	19	16	13	14	12	10	8	5	5	4	0	1342
2020/2/23	345	417	98	84	96	66	62	25	23	22	19	16	14	14	12	10	8	5	5	4	0	1345
2020/2/24	346	417	98	84	97	66	62	25	23	22	19	16	14	14	12	10	8	5	5	4	0	1347
2020/2/25	346	417	98	84	97	66	62	25	23	22	19	16	14	14	12	10	8	5	5	4	0	1347
2020/2/26	346	417	98	84	97	66	62	25	23	22	19	16	14	14	12	10	8	5	5	4	0	1347

**Table 2:** The cumulative recovered cases of COVID-19 in various cities of Guangdong Province (case).

Date	Guangzhou	Shen zhen	Zhu hai	Fo shan	Dong guan	Zhongshan	Hui zhou	Shan tou	Jiangmen	Zhan jiang	Zhao qing	Mei zhou	Yang jiang	Mao ming	Qing yuan	Shao guan	Jie yang	Shan wei	Chao zhou	He yuan	Yunfu	Total
2020/1/19	0	0	0	0	0	0	0	0	0	0	0	0	0	0	0	0	0	0	0	0	0	0
2020/1/20	0	0	0	0	0	0	0	0	0	0	0	0	0	0	0	0	0	0	0	0	0	0
2020/1/21	0	0	0	0	0	0	0	0	0	0	0	0	0	0	0	0	0	0	0	0	0	0
2020/1/22	0	0	0	0	0	0	0	0	0	0	0	0	0	0	0	0	0	0	0	0	0	0
2020/1/23	0	2	0	0	0	0	0	0	0	0	0	0	0	0	0	0	0	0	0	0	0	2
2020/1/24	0	2	0	0	0	0	0	0	0	0	0	0	0	0	0	0	0	0	0	0	0	2
2020/1/25	0	2	0	0	0	0	0	0	0	0	0	0	0	0	0	0	0	0	0	0	0	2
2020/1/26	0	2	0	0	0	0	0	0	0	0	0	0	0	0	0	0	0	0	0	0	0	2
2020/1/27	0	4	0	0	0	0	0	0	0	0	0	0	0	0	0	0	0	0	0	0	0	4
2020/1/28	0	4	0	0	0	0	1	0	0	0	0	0	0	0	0	0	0	0	0	0	0	5
2020/1/29	0	4	0	0	0	0	1	0	0	1	0	0	0	0	0	0	0	0	0	0	0	6
2020/1/30	1	4	1	0	0	0	1	0	0	1	0	0	0	0	0	0	0	0	0	0	0	8
2020/1/31	3	4	1	0	0	0	1	0	0	1	1	0	0	0	1	0	0	0	0	0	0	12
2020/2/1	3	5	1	1	0	0	1	0	0	1	1	0	0	0	1	0	0	0	0	0	0	14
2020/2/2	3	5	1	1	0	0	1	0	0	1	1	0	0	0	2	0	0	0	0	0	0	15
2020/2/3	3	10	1	1	0	0	1	0	0	1	1	0	0	0	2	1	0	0	0	0	0	21
2020/2/4	7	13	1	1	0	3	1	1	0	1	1	0	0	0	2	1	0	0	0	0	0	32
2020/2/5	13	16	2	1	1	4	1	3	0	4	1	0	0	0	2	1	0	0	0	0	0	49
2020/2/6	20	22	3	2	1	6	1	3	1	4	1	0	0	0	2	1	0	1	0	0	0	68
2020/2/7	27	31	3	4	1	11	1	3	1	4	1	4	1	1	2	1	0	1	0	0	0	97
2020/2/8	36	39	4	4	1	14	1	5	1	6	1	4	1	1	3	2	1	1	0	0	0	125
2020/2/9	42	46	4	4	3	14	1	6	1	6	3	4	1	1	3	2	1	1	0	0	0	143
2020/2/10	49	56	11	4	4	16	4	6	2	7	4	4	1	1	5	3	2	1	0	1	0	181
2020/2/11	70	66	17	15	7	21	4	8	2	9	7	4	1	2	5	3	3	1	0	1	0	246
2020/2/12	78	81	21	17	7	22	7	8	2	9	7	5	5	2	5	3	3	1	0	1	0	284
2020/2/13	93	94	24	18	7	26	14	9	2	11	7	7	5	2	5	3	3	1	0	1	0	332
2020/2/14	106	104	34	23	10	31	18	9	2	11	7	7	5	3	7	3	4	1	0	1	0	386
2020/2/15	121	115	38	23	12	34	26	11	2	11	8	7	7	3	7	4	4	1	1	1	0	436
2020/2/16	131	131	38	23	14	39	26	13	3	11	8	7	7	3	7	5	4	1	1	1	0	473
2020/2/17	142	152	41	23	19	40	34	14	5	11	8	7	9	3	9	5	4	2	1	1	0	530
2020/2/18	148	163	41	27	24	40	36	18	8	11	8	10	9	6	9	5	4	2	3	1	0	573
2020/2/19	157	182	45	33	26	40	38	20	9	11	8	10	9	6	9	5	4	2	4	1	0	619
2020/2/20	172	199	49	34	27	42	41	20	9	12	8	10	10	6	9	5	4	2	4	1	0	664
2020/2/21	190	222	52	34	31	46	43	20	9	12	8	11	10	6	9	6	4	2	4	1	0	720
2020/2/22	196	226	52	34	31	49	46	21	9	12	10	11	10	6	10	6	4	2	4	1	0	740
2020/2/23	204	237	54	34	36	51	46	21	9	14	10	11	10	6	10	6	4	2	4	1	0	770
2020/2/24	210	249	56	34	40	53	49	21	10	14	10	11	10	7	12	6	5	4	4	2	0	807
2020/2/25	222	262	59	37	40	55	49	21	10	14	10	11	10	7	12	7	5	4	4	2	0	841
2020/2/26	230	271	61	38	43	55	52	21	11	14	13	12	10	7	12	7	5	4	4	3	0	873

## 4 The critical control policies in Guangdong province

Guangdong is a major province with economy and population movement, which plays an important role in stemming the spread of the COVID-19 epidemic in China. On January 19, 2020, the first imported case of COVID-19 was confirmed in Guangdong Province.^[7]^ On January 23, 2020, Guangdong Province decided to activate the first-level response to public health emergencies.^[8]^ Subsequently, a series of control policies were implemented, which as summarized in Table 3.

**Table 3:** The critical control policies in Guangdong Province.

SN	Date	Policies	Key elements
1	Jan 23	Activating the first-level response to public health emergencies	Governments at all levels took correspond emergency prevention and control measures.
2	Jan 27	32 consecutive press conferences were held on epidemic prevention and control	Reporting the daily epidemic situation, responding to social concerns and guiding public opinion.
3	Jan 28	Grid investigation was carried out in the whole province	Based on the community governance concept of grid management, establishing the village “two committees” system of epidemic prevention and control, setting up a working team with full-time and part-time staff. Clarifying the responsibility to the people so as to ensure all prevention and control measures were effectively implemented.
4	Jan 30	Taking the lead in covering the treatment costs of suspected patients in basic medical insurance	Increasing the intensity of medical security. The suspected patients were paid in advance by government finance when they sought medical treatment.
5	Feb 8	TJQW was used in 30 designated hospitals	Sixteen pieces of traditional Chinese medicine in the prescription were extracted and then mixed into granules, which had obvious effect on the treatment of COVID-19 patients with mild and moderate symptoms.
6	Feb 9	Launching a registration and report system for the sale of fever and cough drugs	Making full use of the advantages of retail pharmacy network to comprehensively screen patients with fever and cough and effectively control the source of infection.

### 4.1 Guangdong province activated the first-level response to public health emergencies firstly in China

On January 23, 2020, Guangdong Province launched the first-level response to public health emergencies. The related agencies should response to the epidemic, and nonincident areas should also take emergency response measures.

#### 4.1.1 People's governments at all levels

People's governments at all levels should coordinate relevant units to response public health emergencies, such as mobilizing all kinds of personnel, materials, means of transportation and related facilities in the administrative area. Based on the epidemic situation, delimiting and blocking epidemic areas, suspending gathering activities, implementing health quarantine in traffic stations and entry-exit ports, releasing epidemic information in time and guaranteeing the supply of materials to maintain social stability.

#### 4.1.2 Health administrative department

Health Administrative Department should propose a response level to initiate emergency treatment of public health emergencies, release information and reporting in time, formulate technical standards for newly discovered infectious diseases and popularize health knowledge to improve public health awareness.

#### 4.1.3 Medical institutions

Medical Institutions should do well in patient reception, admission, and transfer, timely exclude or confirm suspected patients, and prevent cross-infection and pollution. They should also assist CDCs to do specimen collection and epidemiological investigations, and do scientific research and international exchanges to speed up the search for the source and diagnosis of the cause.

#### 4.1.4 Disease prevention and control agency

Disease prevention and control agencies at all levels should do well in information collection, reporting, and analysis of public health emergencies. In addition, they also investigate on epidemiology and laboratory testing, organize technical training, carry out scientific research, and international exchanges.

#### 4.1.5 Health supervision agency

Health Supervision Agency should supervise the implementation of various measures to deal with public health emergencies in medical institutions and CDCs. Furthermore, they also should carry out health supervision and law enforcement inspections on food hygiene, environmental hygiene and occupational hygiene, and assist the health administrative department to investigate and handle illegal acts in responding to public health emergencies.

#### 4.1.6 Entry-exit inspection and quarantine agency

Entry-exit Inspection and Quarantine Agency should mobilize the technical force to cooperate with the local health administrative department for responding to public health emergencies at the port, and report the situation in time.

#### 4.1.7 Emergency response measures in nonincident areas

Areas where public health emergencies have not occurred should analyze the possibility of the affected areas base on the nature, characteristics, regions and development trends of other areas and do relevant preparations to response public health emergencies.

### 4.2 Holding press conferences to cope with the COVID-19 epidemic

Between January 21, 2020 and February 26, 2020, the Guangdong Provincial Information Office held a total of 32 press conferences for response to the COVID-19 epidemic.^[9]^ The key press conferences are as follows.

On January 21, 2020, a special press conference on anti-epidemic was held in Guangdong Province, which was also the earliest provincial antiepidemic press conference in China. Academician Nanshan Zhong, who just confirmed the COVID-19 can be transmitted from human-to-human in Wuhan city and professionally analyzed the current situation of the COVID-19 epidemic, reminded people not to relax their vigilance. On January 27, the second press conference was held in Guangdong Province. The head of the Information Office of the Provincial Government indicated that a press conference would be held every day, and all cities would not be locked down, but inspection and epidemic prevention stations would set up in some places.

At the press conference on January 28, the Guangdong provincial government announced that excluding relevant enterprises, all kinds of enterprises went back to work no earlier than 0:00 am on February 9, and extended school closures. On February 11, the press conference pointed out that the number of new confirmed cases has been declining since February 3, so the epidemic data changed to report every morning as of 0:00 am on the previous day, and the new data from 0:00 am to 12:00 pm were no longer reported. On February 24, the press conference indicated that Guangdong Province decided to adjust the first level response to public health emergencies to the second level response. But adjusting the response level did not represent the arrival of a turning point. The uncertainty and risk of epidemic is still great.

### 4.3 Carrying out grid investigation in Guangdong Province

On January 28, 2020, based on the situation of the epidemic, the Office of Guangdong Epidemic Prevention and Control Headquarters issued the “Emergency Notice on the Comprehensive Implementation of the ‘Four Ones’ Emergency Response Mechanism and the Implementation of the ‘Grid’ Epidemic Prevention and Control Work” for controlling the spread of the COVID-19 epidemic.

On January 31, 2020, grid staff of various communities carried out a grid survey on 18,654 units, including a total of 237,800 people and medical observation of 120,000 people.^[10,11]^ The grid epidemic prevention and control was comprehensively implemented in villages throughout Guangdong Province, so as to ensure close contacts were isolated and people who returned to Guangdong Province from affected areas were not missed.

### 4.4 Taking the lead in covering the treatment expenses of suspected patients in basic health insurance

On January 23, 2020, the Provincial Medical Insurance Bureau and the Provincial Department of Finance jointly suggested that medical security departments at all levels should ensure patients would not be denied treatment because of expenses, and designated medical institutions would not be affected due to the budget management regulations of the total medical insurance.^[12]^

On January 30, 2020, Guangdong Province took the lead in temporarily covering all suspected and confirmed insured patients’ drugs and medical services expenses into the medical insurance fund, and canceled the starting payment standard for inpatients. It was clear that the personal burden would be subsidized by the finances. As of January 30, a total of 1.41 billion Yuan of medical insurance funds was paid to designated medical institutions.

### 4.5 Applying Toujie Quwen granules to 30 designated hospitals

After the COVID-19 outbreak, the Chinese government advocated the therapy with integration of traditional Chinese medicine (TCM) and Western medicine to raise recovery rate and lower case-fatality rate of COVID-19 patients. Tuojie Quwen granules (TJQW, formerly known as pneumonia No. 1 formulation) based on the febrile disease theory of TCM and climate characteristics of Lingnan region, which has achieved good therapeutic results. And TJQW, as a kind of recommendation TCM formulations for COVID-19 treatment, have been mainly used to treat mild to moderate COVID-19 patients to effectively reduce the symptoms of fever, cough, and expectoration.^[13]^

On February 8, 2020, the Guangdong Provincial Drug Administration approved TJQW to use in the 30 designated hospitals.^[14]^ And nondesignated hospitals applying for transfer would be given priority approved by the Provincial Drug Administration.

### 4.6 Launching a registration and report system for the sale of fever and cough drugs

On February 9, 2020, the Provincial Drug Administration proposed that all retail pharmacies would implement a registration and reporting system for the sale of fever and cough drugs from February 9.^[12,15]^ All retail pharmacies must detect body temperature for people, register their information, and complete the “Registration Form for Information on the Purchase of Fever and Cough Medicines in Retail Pharmacies.” Online drug purchases must also be registered. The Provincial Drug Administration required that third-party platforms of Internet drug sales should do well in registering and reporting.

Additionally, to those people who had fever symptoms, came from Hubei or Wenzhou and have been to Hubei, Wenzhou and other areas with high epidemic rates within 14 days, the retail pharmacies’ staff should persuade them to do personal protection and go to medical institutions in time, and report to the local “Joint Defence Team” (composed of the local neighborhood committees, general practitioners, and community police).

## 5 The effect of control policy on coping with COVID-19 in Guangdong province

**Figure 1:**
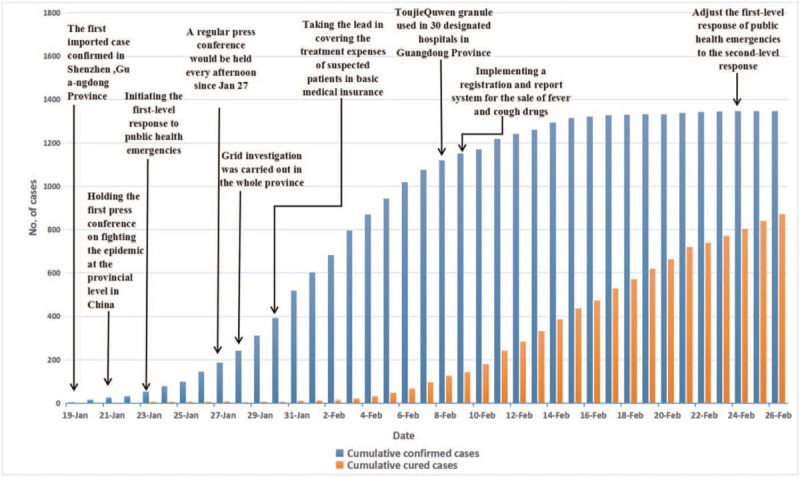
The COVID-19 epidemic data and control policy in Guangdong Province.

Figure 1 shows that from January 19 to February 7 the number of confirmed cases increased rapidly in Guangdong Province from 1 to 1075, while the number of recovered cases increased steadily from 0 to 97. From February 8 to 14 the number of confirmed cases increased steadily from 1120 to 1294, while the number of recovered cases increased rapidly from 125 to 386. From February 15 to 26 the increase number of confirmed cases was a small in magnitude from 1316 to 1347, while the number of recovered cases increased faster than before from 436 to 873.

**Figure 2:**
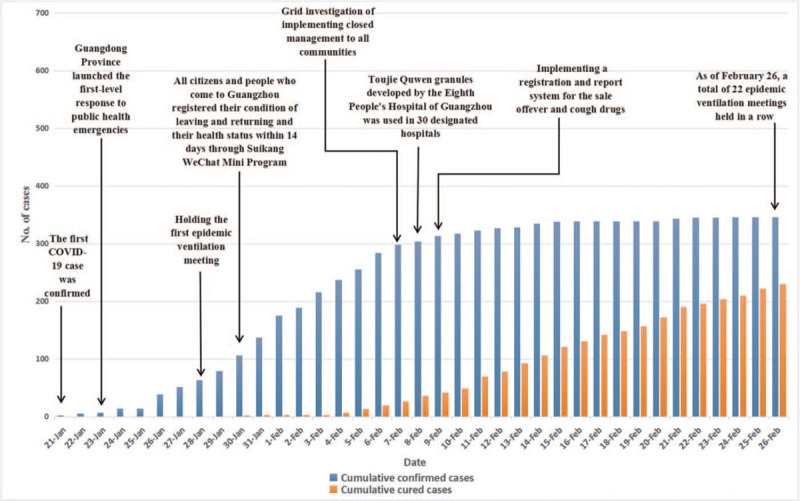
The COVID-19 epidemic data and control policy in Guangzhou.

Figure 2 presents that from January 21 to February 7, 2020 the number of confirmed cases rose rapidly in Guangzhou from 1 to 298, while the increase of recovered cases was small in magnitude from 0 to 27. From February 8 to 14 the number of confirmed cases rose gradually from 304 to 335, while the number of recovered cases rose rapidly from 36 to 106. From February 15 to 26 the increase of confirmed cases was small in magnitude from 338 to 346, while the number of recovered cases rose faster than before from 121 to 230.

**Figure 3:**
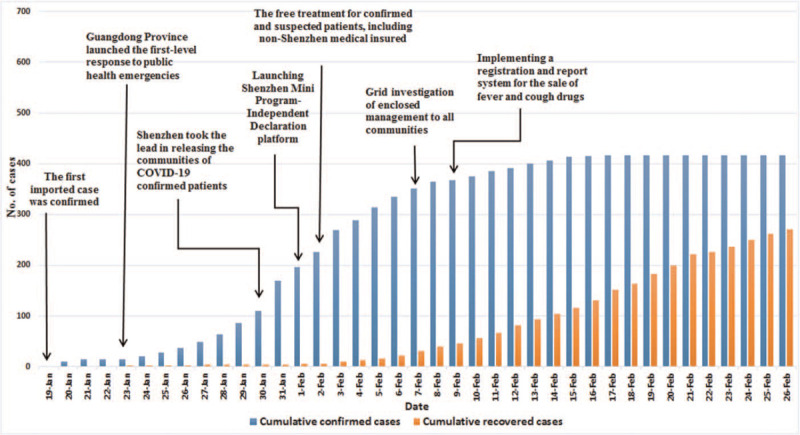
The COVID-19 epidemic data and control policy in Shenzhen.

Figure 3 shows that from January 19 to February 7, 2020 the number of confirmed cases increased rapidly in Shenzhen from 1 to 351, while the increase of recovered cases was a small in magnitude from 0 to 31. From February 8 to 14 the number of confirmed cases increased steadily from 364 to 406, while the number of recovered cases increased sharply from 39 to 104. From February 15 to 26 the increase of confirmed cases was a small in magnitude from 414 to 417, while the number of recovered cases increased faster than before from 115 to 271.

Overall, the change trend of epidemic data is basically the same in Guangdong Province, Guangzhou city and Shenzhen city. From January 19 to February 7 2020, the number of confirmed cases increased steeply, but the increase of recovered cases was a small in magnitude. From February 8 to 14, the number of confirmed cases increased gradually, and the number of recovered cases increased steeply. From February 15 to 26 the increase of confirmed cases was a small in magnitude, but the number of recovered cases increased faster than before.

## 6 Discussion

The results of a paper published by academician Nanshan Zhong research group showed that median incubation period of COVID-19 was 3.0 days (range, 0 to 24.0 days).^[6]^ This result indicated that control the COVID-19 outbreak would be taken a period of time. On January 19, 2020, the first imported case was found in Guangdong Province. As the incubation period of the COVID-19 was 0 to 24 days, the confirmed cases appeared a turning point on February 15 after the implementation of control policies. This shows that the policies of coping with COVID-19 achieved remarkable results in Guangdong Province.

### 6.1 The policies of fighting COVID-19 achieved remarkable effects in Guangdong Province

Figure 1 shows that from January 19 to February 7, 2020, the number of confirmed cases increased rapidly, from 1 to 1075. In this period, Guangdong Province initiated the first-level response to public health emergencies; held press conference; carried out grid investigation; and took the lead in covering the treatment expenses of suspected patients in basic medical insurance. These 4 policies have played an important role in the large-scale examination, screening, and investigation of the COVID-19 patients, so the number of confirmed cases had continued to increase rapidly. From February 8 to 14, the number of confirmed cases increased steadily from 1120 to 1294. Guangdong Province continued to apply TJQW to 30 designated hospitals and launch a registration and report system for the sale of fever and cough drugs. These 2 policies have further controlled the COVID-19 epidemic. From February 15 to 26, the increase of confirmed cases was a small in magnitude from 1316 to 1347. On February 24, the first level response to public health emergencies adjusted to the second level response in Guangdong Province. This suggests that the 6 policies had a critical effect on controlling the spread of the COVID-19 epidemic.

Figure 1 shows that from January 19 to February 7, 2020, the number of recovered cases increase slowly from 0 to 97. From February 8 to 14, the number of recovered cases increased rapidly from 125 to 386. Especially since February 8, the TJQW were used in 30 designated hospitals and the clinical treatment course of TJQW is 6 days (or more).^[16]^ Thus the number of recovered cases increased faster than before, from 436 on February 15 to 873 on February 26. This suggests that the TJQW played a key role in the treatment of COVID-19 patients and improved the cure rate.

### 6.2 The policies of fighting COVID-19 achieved remarkable effects in Guangzhou

Figure 2 shows that from January 21 to February 7, 2020, the number of confirmed cases increased rapidly in Guangzhou from 1 to 298. In this period, Guangzhou government held epidemic ventilation meetings and carried out grid investigation to contain the spread of COVID-19 epidemic. Grid investigation was that all citizens and people who come to Guangzhou registered their departure and return and health status within 14 days through Suikang WeChat Mini Program, as well as the implementation of closed management to all communities. These 2 policies sped up the large-scale examination and screening of the COVID-19 patients, so the confirmed cases continued to increase rapidly. Especially on February 7, all communities implemented closed management. From February 8 to 14, the number of confirmed cases increased gradually from 304 to 335. In this period, Guangzhou government implemented a registration and reporting system for the sale of fever and cough drugs to further screen patients, so the epidemic could be effectively prevented and controlled. From February 15 to 26, the increase of confirmed cases was a small in magnitude from 338 to 346. This shows that the 2 control policies exhibited significant effects on COVID-19 control.

Table 2 shows that from January 21 to February 4, 2020, the number of recovered cases was less than 10, from 0 to 7. Figure 2 shows that from February 5 to 14, the number of recovered cases increased rapidly from 13 to 106. Especially on February 8, TJQW was approved to be used in 30 designated hospitals, so from February 15 to 26, the recovered cases increased rapidly from 121 to 230. This shows that TJQW sped up the treatment of COVID-19 patients and improved the recovery rate.

### 6.3 The policies of fighting COVID-19 achieved remarkable effects in Shenzhen

Figure 3 shows that from January 19 to February 7, 2020, the number of confirmed cases rose rapidly in Shenzhen from 1 to 351. Shenzhen, as the first city to confirm imported cases in Guangdong Province, had accelerated the early detection, early isolation and early treatment of confirmed patients by implementing the policies of free treatment for suspected and confirmed patients including non-Shenzhen medical insured and grid investigation. The grid investigation included: releasing the activity areas of confirmed patients, all citizens’ registering information through Shenzhen WeChat Mini Program, and implementing closed management to all communities. The close tracking and investigation of patients had led to the confirmed cases increased rapidly, which was conducive to quickly identify the source of infection and effectively prevent further spread of the epidemic. From February 8 to 14, the number of confirmed cases rose steadily from 364 to 406, because a registration and report system for the sale of fever and cough drugs was implemented for speeding up diagnosis cases. From February 15 to 26, the increase number of confirmed cases was a small in magnitude from 414 to 417. This shows that the control policies had effectively contained the spread of the COVID-19 epidemic.

Table 2 shows that from January 19 to February 3, 2020, the number of cured cases remained below 10. Figure 3 shows that from February 4 to 26, the number of recovered cases continued to grow steadily from 13 to 271. This shows that from January 19 to February 9, the control policies, “implementing free treatment for suspected and confirmed patients” and “including non-Shenzhen medical insured and grid investigation,” had accelerated the early detection and treatment of confirmed patients, which resulted in a rapid increase in the number of cured cases.

### 6.4 The control policies of Guangzhou and Shenzhen played a key role in fighting COVID-19 in Guangdong Province

Figures 1–3 show that the number of COVID-19 patients in Guangdong Province was closely related to Guangzhou and Shenzhen. The control policies of Guangzhou and Shenzhen not only directly affected the change in the number of COVID-19 cases in Guangzhou and Shenzhen, but also indirectly affected the number of COVID-19 cases in Guangdong Province. For example, on February 7, 2020, Guangzhou and Shenzhen implemented closed management to all communities, the confirmed cases appeared a turning point, and then a turning point was also appeared in Guangdong Province. In terms of the recovered cases, on February 5, 2020, there was a turning point in Guangzhou and Shenzhen, and then a turning point was appeared in Guangdong Province. This indicates that the control policies of Guangzhou and Shenzhen played a key role in controlling the COVID-19 epidemic in Guangdong Province, and the trend of the number of confirmed cases and recovered cases was consistent in Guangzhou and Shenzhen.

## 7 Conclusions

In conclusion, the study showed that Guangdong Province issued the following 6 policies: initiating the first-level response to public health emergencies; holding 31 routine press conferences to cope with COVID-19 in a row; taking the lead in covering the treatment expenses of suspected patients in basic medical insurance; carrying out grid investigation; applying TJQW to 30 designated hospitals; and launching a registration and reporting system for the sale of fever and cough drugs. These 6 policies have played a critical role in containing the spread of the COVID-19 epidemic. In particular, these 2 policies, implementing closed management to all communities in Guangzhou and Shenzhen and applying TJQW to 30 designated hospitals, made a substantial contribution to the COVID-19 epidemic control. Therefore, these 6 policies played a critical role in effectively controlling of COVID-19 in China and provided valuable experience for global currently fighting the COVID-19 pandemic.

## Acknowledgments

The authors thank all study participants who have been involved and contributed to the procedure of data collection.

## Author contributions

**Conceptualization:** Leiyu Shi, Gang Sun.

**Data curation:** Yuyao Zhang, Xiaohan Wang.

**Supervision:** Gang Sun.

**Writing – original draft:** Haiqian Chen.
